# A Case of Combination of IgA Nephropathy and Interstitial Nephritis After COVID-19 Vaccination

**DOI:** 10.7759/cureus.52981

**Published:** 2024-01-26

**Authors:** Yuki Yoshida, Kenta Torigoe, Ryosuke Sakamoto, Shinichi Abe, Kumiko Muta, Hiroshi Mukae, Tomoya Nishino

**Affiliations:** 1 Nephrology, Nagasaki University Hospital, Nagasaki, JPN; 2 Respiratory Medicine, Nagasaki University Graduate School of Biomedical Sciences, Nagasaki, JPN

**Keywords:** glomerular nephritis, severe acute respiratory syndrome coronavirus 2, covid-19 vaccine, tubulointerstitial nephritis, iga nephropathy (igan)

## Abstract

A 66-year-old male presented with renal dysfunction. At the time of presentation, his serum creatinine (sCr) was 2.55 mg/dL, estimated glomerular filtration rate (eGFR) was 20.93 ml/min/1.73 m^2^, urinary red blood cell (RBC) was 30-49/high power field, and urine protein-creatinine ratio was 0.43 g/gCr. The patient had no urinalysis abnormalities or renal dysfunction within the year prior to presentation but had gross hematuria after the third and fourth coronavirus disease 2019 (COVID-19) vaccinations. Therefore, immunoglobulin A nephropathy (IgAN) was suspected and a percutaneous renal biopsy was performed. Renal pathology confirmed IgAN and interstitial nephritis and glucocorticoid therapy was initiated. Glucocorticoids improved renal function, and microscopic hematuria resolved. Although previous reports have shown that the COVID-19 vaccine induces various renal diseases, complications associated with these two renal diseases are rare. In this case, while IgAN was suspected based on episodes of gross hematuria after vaccination, renal biopsy confirmed it and also revealed interstitial nephritis.

## Introduction

The coronavirus disease 2019 (COVID-19) pandemic caused by severe acute respiratory syndrome coronavirus 2 (SARS-CoV-2) virus infection remains a global concern. Although effective treatments for COVID-19 have been developed, it can still be fatal. Traditional and mRNA vaccines against COVID-19 have been reported to effectively reduce the infection rate and risk of severe infection and are now in widespread use [1,2]. Adverse events associated with mRNA-based COVID-19 vaccines commonly include injection site pain and fatigue, which are transient and do not constitute major problems [3]. However, renal-related adverse events can also occur after COVID-19 vaccination and can sometimes be severe. Previous reports have shown that COVID-19 vaccination induces renal diseases such as minimal change disease, antineutrophil cytoplasmic antibody (ANCA)-related vasculitis, immunoglobulin A nephropathy (IgAN), and interstitial nephritis [4]. Although reports of these renal-related adverse events have accumulated over the years since the introduction of mRNA-based COVID-19 vaccines, most involve only one renal disease and rarely multiple renal diseases. Here, we present a case of combined IgAN and interstitial nephritis following mRNA-based COVID-19 vaccination.

## Case presentation

A 66-year-old man with renal dysfunction was referred to our department. The patient had a history of hypertension for five years, and an estimated glomerular filtration rate (eGFR) of 69.9 ml/min/1.73 m^2^ (normal renal function) one year prior to referral, with no abnormal urinalysis. The patient had received the third and fourth mRNA-based COVID-19 vaccines seven and two months before referral, respectively, with fever and gross hematuria. Both fever and gross hematuria resolved spontaneously. However, the patient was referred to our department because blood tests by his family physician revealed renal dysfunction with an eGFR of 20.7 ml/min/1.73 m^2^.

At the time of referral, there was no gross hematuria or other symptoms. He was 162 cm tall and weighed 78.7 kg. His body temperature was 36.7 °C, blood pressure was 144/73 mmHg, pulse 58/min, and oxygen saturation (SpO_2_) 97% (room air). Physical examination revealed no obvious abnormalities such as skin rash, arthralgia, or edema. Blood tests revealed renal dysfunction with serum creatinine (sCr) 2.55 mg/dL and eGFR 20.93 ml/min/1.73 m^2^. Immunological tests were negative for antinuclear antibodies (ANA), ANCA, and glomerular basement membrane (GBM) antibodies. There were no complement or immunoglobulin abnormalities with levels of complement components 3 (C3), and 4 (C4) 113.5 and 22.7 mg/dL, respectively, with total hemolytic complement (CH50) 43.2 CH50/mL, and IgG, IgA, and IgM of 1473, 322, and 154.3 mg/dL, respectively. Urinalysis revealed microscopic hematuria with urinary red blood cells (RBC) of 30-49/ high-power field (HPF) and white blood cells (WBC) of < 1 /HPF. Urinary protein and β2-microglobulin were elevated at urinary protein 0.43 g/gCr and β2-microglobulin 2945 μg/L.

**Figure 1:**
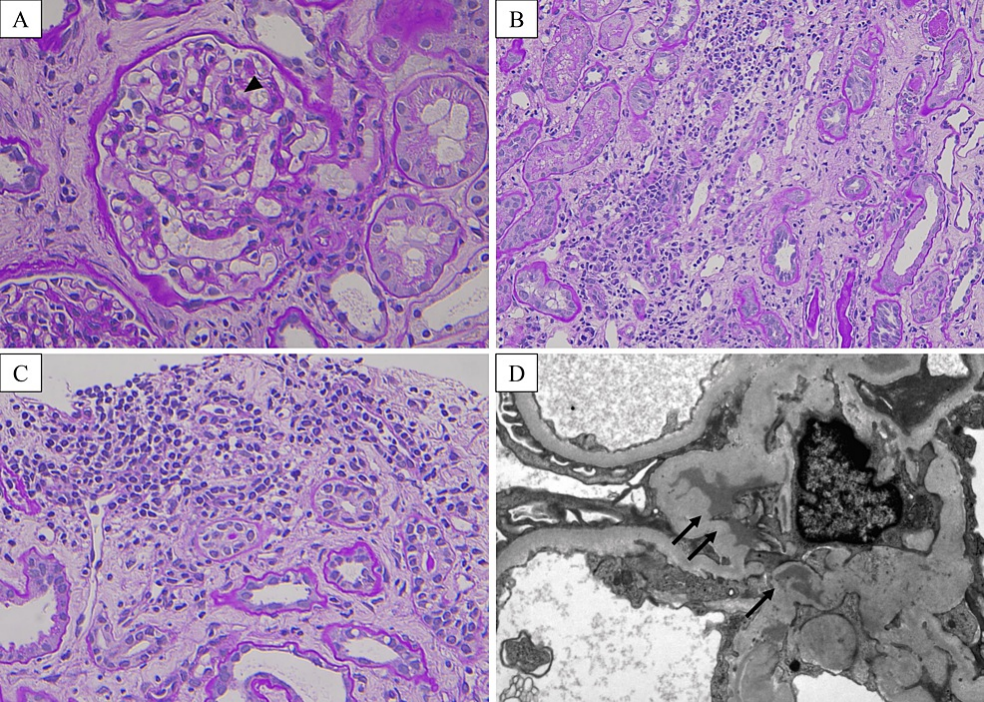
Renal pathology findings of light and electron microscopy. (A) Mild mesangial hypercellularity was observed in glomeruli (arrowhead) (PAS staining). (B, C) The tubulointerstitium showed inflammatory cell infiltration and tubulitis (PAS staining). (D) Electron microscopy showed electron-dense deposits in mesangial area (arrow) PAS: periodic acid-Schiff

Based on the history of gross hematuria after the COVID-19 vaccination, IgAN was suspected, and the patient was admitted on the fifth day of referral and underwent percutaneous renal biopsy. Renal pathology revealed that 34.8% of glomeruli exhibited global glomerulosclerosis, and 30.4% exhibited mesangial hypercellularity. There were no active lesions such as crescent formation or endocapillary proliferation (Figure 1A), whereas the tubular interstitium was partially edematous and infiltrated with inflammatory cells, and tubulitis was observed (Figure 1B, 1C). In addition, tubulointerstitium showed moderate involvement with fibrocellular change in approximately 30% and tubular atrophy in around 20% of the cortex. Arterioles demonstrated moderate sclerosis with hyaline change in about 20% of arterioles.

**Figure 2:**
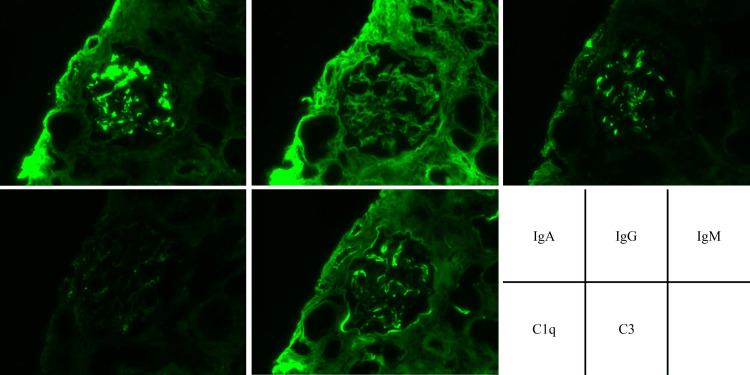
Renal pathology findings of immunofluorescence microscopy IgA (3+) and C3 (2+) were predominantly positive in the mesangial area. IgM (1+) was weakly positive in the same area.

Immunofluorescence staining showed predominant positivity for IgA (3+) and C3 (2+) in the mesangial area (Figure 2). Deposition in the mesangial area was revealed by electron microscopy (Figure 1D).

**Figure 3:**
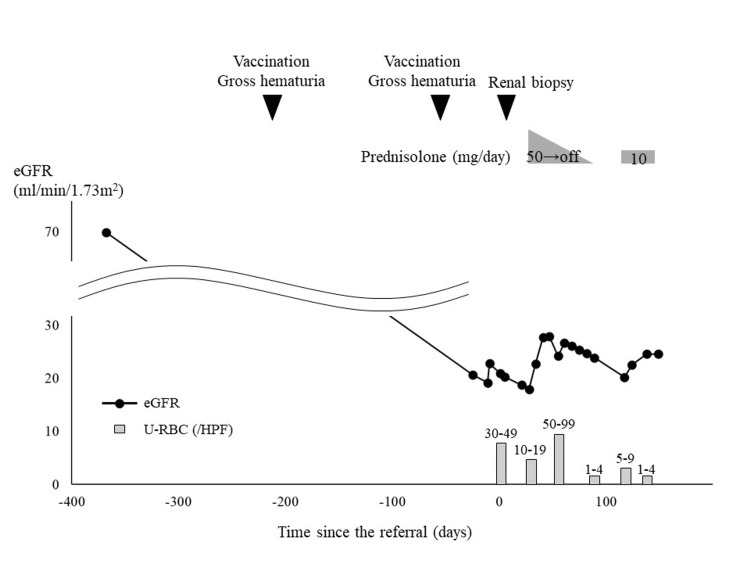
The clinical course of the patient eGFR: estimated glomerular filtration rate; HPF: high power field; U-RBC: urinary red blood cell

Based on these results, the patient was diagnosed with IgAN and tubulointerstitial nephritis. The MEST-C score was M1E0S0T1-C0. The patient's history of autoimmune diseases and medications was investigated as a possible cause of interstitial nephritis; however, there was no obvious causative autoimmune disease or drug, except for the COVID-19 vaccine. Therefore, we surmised that the COVID-19 vaccine may cause interstitial nephritis. The COVID-19 vaccine was also considered the cause of IgAN because of gross hematuria after vaccination. Based on the renal pathology findings, there were no active lesions of IgAN, but active lesions of tubulointerstitial nephritis, such as tubulitis, were present. The patient was discharged from our hospital after a renal biopsy and readmitted to our department on day 28 of being referred for the treatment of interstitial nephritis. The patient was prescribed prednisolone 50 mg/day (Figure 3).

After initiation of treatment, the eGFR improved to 27.92 ml/min/1.73 m^2^, and the urinary RBC improved to 1-4/HPF. Prednisolone was discontinued on day 89 of the referral. However, renal function deteriorated again after discontinuation of prednisolone and it was restarted at 10 mg/day on day 117 of referral. Since then, his eGFR has been stable at about 24 ml/min/1.73 m^2^, and he has been on maintenance therapy with prednisolone.

## Discussion

The present case involved a combination of IgAN and interstitial nephritis after two COVID-19 vaccinations. Unlike previous reports, this case is unique in that the mRNA-based COVID-19 vaccine caused two types of nephritis.

Various renal diseases have been reported to be caused by the mRNA-based COVID-19 vaccine and IgAN is the most common amongst them. The mechanism of vaccine-induced IgAN is hypothesized to involve the production of excessive antiglycan antibodies that cross-react with galactose-deficient IgA (Gd-IgA); however, the mechanism has not been fully established [5]. In the present case, no gross hematuria was observed until the second vaccination session. The urinalysis results and renal function were normal, suggesting that the patient did not have renal disease until the second vaccination session. However, gross hematuria was observed after the third and fourth vaccinations, suggesting that the patient developed IgAN after the third vaccination. Normally, even if gross hematuria is observed after COVID-19 vaccination, renal dysfunction does not progress. However, renal function was impaired due to the third and fourth vaccinations in the present case [6]. Unlike previous cases, interstitial nephritis was considered one of the factors contributing to the progression of renal dysfunction in this case. Concerning IgAN with acute renal dysfunction, the differential diagnosis included acute tubular necrosis, crescentic glomerulonephritis, and renal injury due to gross hematuria. A kidney biopsy is the only way to determine the differential diagnosis, which revealed not only IgAN but also tubulointerstitial nephritis in this patient.

**Figure 4:**
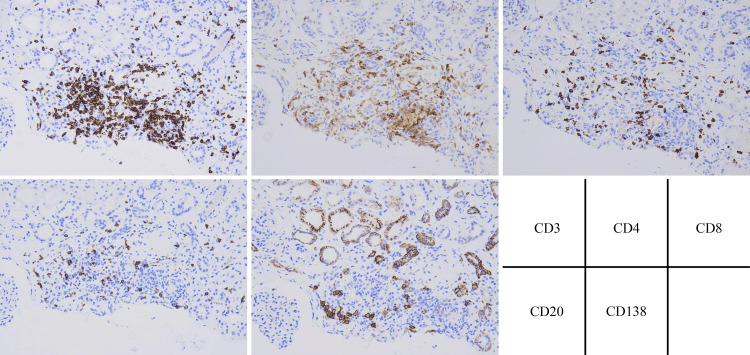
Immunohistochemistry of cell surface markers CD3, CD4, and CD8 were predominantly positive in infiltrated inflammatory cells of the tubulointerstitium.

Tubulointerstitial nephritis and IgAN are known complications of the COVID-19 vaccine [4]. Medical drugs are the most common cause of tubulointerstitial nephritis [7]. In the present case, there were no other autoimmune diseases causing interstitial nephritis; therefore, we considered the tubulointerstitial nephritis to be caused by the COVID-19 vaccine. T cells play a central role in drug-induced interstitial nephritis, and immunostaining in this case showed that the inflammatory cells infiltrating the interstitium were mainly T cells (CD3/4/8) (Figure 4) [8]. Therefore, T cells likely play a central role in COVID-19-induced interstitial nephritis, similar to other types of drug-induced interstitial nephritis. In this case, the proliferative glomerular lesions were mild, but inflammatory cells in the interstitium and tubulitis were present and were more associated with the active lesions of interstitial nephritis than in IgAN. The clinical symptoms of interstitial nephritis are fever, arthralgia, and rash; however, these symptoms are not always present. In this case, there were no clinical symptoms characteristic of interstitial nephritis other than transient fever after vaccination, making it difficult to diagnose based on the clinical course [7]. Even if IgAN is suspected due to gross hematuria after vaccination, renal biopsy may be an effective option for investigating other renal disease complications when progressive renal dysfunction is observed.

Previous cases of combined IgAN and interstitial nephritis after mRNA-based COVID-19 vaccination are shown in Table 1 [9-11]. All patients showed renal dysfunction and a renal biopsy was performed because of renal dysfunction or abnormal urinalysis. All patients were treated with glucocorticoids, but the sCr level of one patient did not improve. Although there is no established treatment for patients with a combination of IgAN and interstitial nephritis, glucocorticoid is recommended for the presence of active lesions in either IgAN or interstitial nephritis [7,12]. Glucocorticoids have been shown to improve renal function in patients with interstitial nephritis that developed after COVID-19 vaccination [6]. In the present case, the renal pathology showed that the patient had active interstitial nephritis. After the initiation of glucocorticoids, renal function improved, albeit only partially. The reason for this is believed to be that the period from the onset of gross hematuria after the third vaccination to the commencement of glucocorticoid treatment was lengthy, resulting in the development of chronic renal lesions. Renal pathology actually showed moderate interstitial fibrosis.

**Table 1:** Summary of published cases with combined IgAN and interstitial nephritis treated with glucocorticoids after COVID-19 vaccination AKI: acute kidney injury; F: female; GC: glucocorticoid; HPF: high-power field; M: male; RBC: red blood cell; sCr: serum creatinine; COVID-19: coronavirus disease 2019; IgAN: immunoglobulin A nephropathy

Authors	Case	Age	Sex	Onset after dose	symptoms	Baseline sCr (mg/dL)	Laboratories					
							Preceding			Follow-up		
														sCr (mg/dL)	Urine RBC (/HPF)	Urine protein (g/gCr)	sCr (mg/dL)	Urine RBC (/HPF)	Urine protein (g/gCr)
Present Case	1	66	M	3^rd^	Gross hematuria	0.86	2.55	30-49	0.43	2.19	1-4	0.2
Klomjit et al. [9]	2	44	M	1^st^	AKI	1.1	2.5	21-30	14	3.6	3-10	5.6
Czerlau et al. [10]	3	38	F	2^nd^	Proteinuria	0.86	0.98	Unknown	0.61	0.9	Unknown	Unknown
Czerlau et al. [10]	4	35	F	2^nd^	AKI	0.56	1.13	Unknown	2	1	Unknown	Unknown
Hishida et al. [11]	5	69	M	1st	AKI	0.8	6.5	5-9	1.0	3.0	Unknown	Unknown

This is an interesting case of two different types of nephritis associated with COVID-19. Since there was no history or other possible causes, we concluded that nephritis was associated specifically with the COVID-19 mRNA vaccine. However, similar cases are rare, and it is difficult to conclude whether the COVID-19 vaccine induces two types of nephritis at the same time. At the same time, we expect that further discussions regarding the mechanism of the disease and the establishment of treatment will result if similar cases are reported in the future.

## Conclusions

This case involved a combination of IgAN and interstitial nephritis likely caused by the COVID-19 vaccine. Renal function was partially improved by glucocorticoids, although renal dysfunction persisted. Although this is not a common occurrence, the COVID-19 vaccine seems likely to have induced two different types of nephritis at same time. Renal biopsy is necessary for diagnosis and should be undertaken as a matter of urgency in cases of persistent renal dysfunction after COVID-19 vaccination.
